# The prognostic significance of stress hyperglycemic ratio in critically Ill patients with hypertension: A study using the MIMIC-IV database

**DOI:** 10.1371/journal.pone.0352162

**Published:** 2026-07-31

**Authors:** Zirong Li, Yufei Wu, Qian Jin, Taiwei Lou, Yi Kang, Xianbei Wang, Ning Sun, Tian Ni, Zhengchuan Zhu, Miaoran Wang, Qiuyan Li

**Affiliations:** 1 Department of General Medicine, Xiyuan Hospital, China Academy of Chinese Medical Sciences, Beijing, China; 2 Out-of-campus Clinical Medical College (Xiyuan Hospital), Graduate School of Beijing University of Chinese Medicine, Beijing, China; 3 Department of Cardiovascular, Xiyuan Hospital, China Academy of Chinese Medical Sciences, Beijing, China; 4 Xiyuan Hospital, National Clinical Research Center for Chinese Medicine Cardiology, Beijing, China; Sapienza University of Rome: Universita degli Studi di Roma La Sapienza, ITALY

## Abstract

**Background:**

Traditional ABG is susceptible to interference from acute stress and daily fluctuations, making it difficult to accurately assess true acute blood glucose surges. To bridge this gap, we adopted the Stress Hyperglycemia Ratio (SHR), which reflects true acute hyperglycemia by controlling for baseline glucose status. SHR is associated with critical illness and has been shown to associated with in-hospital mortality. However, there is a lack of studies investigating SHR and its prognostic significance in patients with hypertension.

**Methods:**

This study utilized the Medical Information Mart for Intensive Care IV database (MIMIC-IV) to extract patient information. All subjects were divided into four groups based on the quartiles of SHR. Kaplan-Meier (KM) curves were utilized to assess the relationship between SHR and all-cause mortality at 30, 90, 180, and 365 days. The relationship between the SHR index and prognosis was evaluated using restricted cubic spline (RCS) regression and Cox proportional hazards regression. At the same time, subgroup analyses were performed for gender, age, diabetes, myocardial infarction, congestive heart failure, cerebrovascular disease, and paraplegia.

**Results:**

A total of 2,140 participants with essential hypertension were included in the study. The KM curve analysis revealed that elevated levels of the SHR index were significantly associated with an increased risk of all-cause mortality at 30, 90, 180, and 365 days (log-rank *P* < 0.05). Moreover, multivariate analysis revealed that the SHR index remained significantly associated with mortality risk (*P <* 0.001**)**. RCS analysis revealed a nonlinear, inverse U-shaped association between SHR and all-cause mortality (*P* < 0.05). Subgroup analysis showed statistically significant differences in all-cause mortality across gender, age, diabetes, myocardial infarction, heart failure, cerebrovascular disease, and paraplegia.

**Conclusions:**

In critically ill patients with hypertension, a high level of SHR index is associated with all-cause mortality. The SHR index may be a potential prognostic indicator for assessing illness severity in ICU patients with hypertension.

## Introduction

Globally, hypertension is currently the leading cause of cardiovascular disease mortality and increased disease burden. From 1990 to 2019, the number of people aged 30–79 years with hypertension increased from 648 million to 1,278 million [1], and in 2015, approximately 8.5 million deaths were strongly associated with hypertension, with 88% of these occurring in low- and middle-income countries [2].In 2019, the main tertiary risk factor attributable to deaths globally was high systolic blood pressure (SBP), accounting for 19.2% [3]. Adherence to a healthy lifestyle and aggressive medication reduces the risk of cardiovascular disease deaths [4–5]. Patients with hypertension are more likely to be admitted to the ICU and use invasive ventilation [6]. Despite the importance of prognostic indicators in managing critical hypertension, there is a relative lack of research in this area. And it should be noted that when patients with hypertension are admitted to the ICU, they are considered critically ill, which is a separate phenomenon from hypertensive crisis.

Stress hyperglycemia is characterized by elevated admission blood glucose (ABG) and is often seen in critically ill patients. It is estimated that 10% of patients in the intensive care unit (ICU) have experienced it [7,8]. Stress-induced hyperglycemia (SIH) significantly elevates blood pressure by triggering acute physiological changes, including peripheral insulin resistance, enhanced gluconeogenesis, and increased levels of counterregulatory hormones such as catecholamines, cortisol, and cytokines [9–11]. However, traditional ABG assessment is constrained by acute stress and daily blood glucose fluctuations, making it difficult to accurately identify genuine acute blood glucose surges. To bridge this gap, we adopted the Stress Hyperglycemia Ratio (SHR)—a metric calculated from ABG and glycated hemoglobin (HbA1c)—which reflects true acute hyperglycemia by controlling for baseline glucose status, thereby enabling its application in evaluating hypertension severity [12,13].

In recent years, several composite indicators of glucose metabolism, such as the TyG index [14], dynamic patterns of multi-class biomarkers [15], neutrophil count and prognostic nutritional index [16], and the glucose-to-potassium ratio [17]—have been shown to be closely associated with the prognosis of critically ill patients. And clinical studies have demonstrated that SHR is associated with adverse cardiovascular events in patients with diabetes [18], acute coronary syndrome [19,20], heart failure [21,22], ischemic stroke [23], cerebral hemorrhage [24], sepsis [25], and chronic kidney disease [26]. However, there is a lack of studies specifically analyzing SHR in critically ill hypertensive patients, which differs from the research conducted on the aforementioned population. Therefore, this study aims to identify patients at risk early to improve outcomes and reduce mortality by analyzing the prognostic significance of SHR in critically ill hypertensive patients.

## Methods

### Source of data

This study utilized data from the Medical Information Mart for Intensive Care IV database (MIMIC-IV, version 3.0). The MIMIC-IV database is a publicly available clinical database used primarily for medical research. Version 3.0, updated in 2024, adds new data on hospitalizations from 2020 to 2022, increasing the number of patients to 364,627 from 299,712 in version 2.2, and new Out-of-hospital mortality data for up to 1 year after hospitalization or emergency discharge, which helps to study the long-term prognosis of patients. The database contains patient demographic information, clinical diagnosis and treatment information, laboratory indicators, and prognostic information. The data in the database are de-identified to ensure patient privacy, and therefore the study does not need to obtain informed consent from patients to use the data. In accordance with the requirements for access to database resources, we completed the relevant training courses and passed the exam, leading to the Physical Network Certification.

### Study design and population

**Fig 1:**
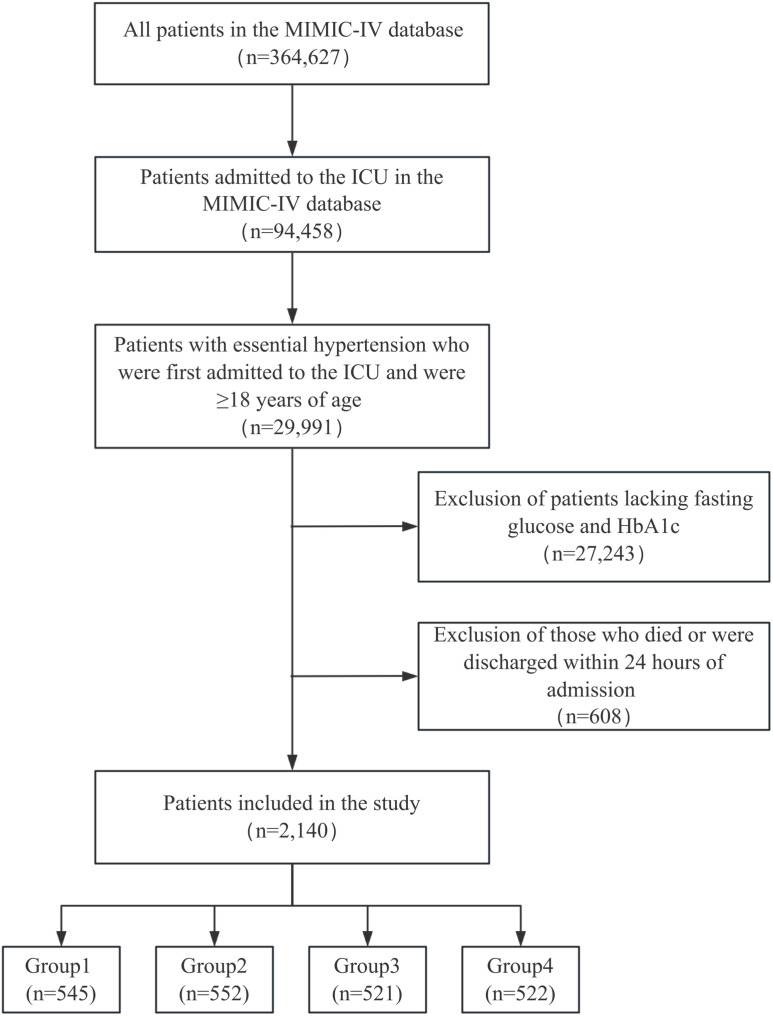
Flowchart for inclusion of number of patients.

The database includes a total of 364,627 patients. Among these patients, there were 94,458 unique ICU stays. Inclusion criteria for the study were patients with essential hypertension who met International Classification of Diseases (ICD) 9 or 10 diagnostic codes. Exclusion criteria were (1) patients not admitted to the ICU for the first time; (2) less than 18 years of age; (3) lacking admission glucose or HbA1c data within 24 hours of admission; (4) those who died or were discharged from the hospital within 24 hours of admission, because too much data on key variables were missing in this population; and (5) patients with incomplete prognostic information. A total of 2,140 patients were finally included (Fig 1), and all subjects were divided into four groups based on the quartiles of SHR (Q1: SHR ≦ 0.86, Q2: 0.86 < SHR ≦ 1.02, Q3: 1.02 < SHR ≦ 1.24, Q4: SHR > 1.24).

### Data extraction

Data extraction was conducted using Navicat Premium (version 17) and SQL, including: (1) demographics; (2) past medical history; (3) laboratory indicators; (4) disease severity scores; (5) treatments; (6) length of hospitalization and (7) outcomes.

### Outcomes

The primary outcome indicator for this study was all-cause mortality at 30 and 365 days, and the secondary outcome indicator was all-cause mortality at 90 and 180 days.

### Definition

The SHR was calculated using the formula: [(admission glucose (mg/dl))/ (28.7 × HbA1c (%)-46.7)]. Admission glucose refers to the first blood glucose value recorded after a patient is admitted to the ICU. Due to database limitations, it was difficult to distinguish whether these readings were fasting. Therefore, all samples were treated as random samples. Since random blood glucose levels are typically slightly higher than fasting levels, the study employed the SHR metric. Combining random blood glucose values with HbA1c, which represents chronic glucose levels, can partially offset the upward bias inherent in non-fasting measurements.

And estimated glomerular filtration rate (eGFR) was calculated as follows: eGFR = 186 × (Scr)^–1.154 × (age)^–0.203 × (0.742 if female). This formula is used to estimate eGFR through the Modification of Diet in Renal Disease equation.

### Covariate selection

When selecting covariates for Cox regression analysis, we based our selection on variables with *P* < 0.001 from the univariate analysis in Table 1 to control for the false-positive rate and retain the strongest prognostic factors. We further screened these variables, taking clinical significance into account, and included those closely associated with glucose metabolism, target organ damage from hypertension, and ICU outcomes, while excluding indicators that might lead to multicollinearity. Although our univariate analysis results did not show differences in age and gender, multiple studies have indicated that there are differences in hemodynamic parameters between genders. Men tend to have higher systolic blood pressure before middle age, while women’s blood pressure also increases significantly with age [27–30]. Regarding comorbidities, we considered that clinical hypertension often coexists with diabetes, and cerebrovascular diseases, so these were included in the study [30]. Specifically, over 50% of patients with diabetes, whether type 1 or type 2, eventually develop hypertension complications [31]. In patients with diabetes, this further increases the incidence of cardiovascular diseases by 2–3 times [32]. Similarly, hypertension has long been a major risk factor for cerebrovascular diseases, as elevated blood pressure can damage cerebral blood vessels through various mechanisms [33,34]. Finally, regarding the selection of laboratory indicators, we focused on biochemical indicators. Studies have shown that biochemical testing can identify patients with hypertension with specific and treatable causes, helping to identify high-risk patients with hypertension based on comorbidities and supporting patient risk stratification. Based on this, we selected the above statistically significant and clinically relevant indicators as covariates [35].

**Table 1:** Baseline characteristics and medication data.

Variables	Total (n = 2140)	Q1 (SHR ≤ 0.86, n = 545)	Q2 (0.86 < SHR ≤ 1.02, n = 552)	Q3 (1.02 < SHR ≤ 1.24, n = 521)	Q4 (SHR > 1.24, n = 522)	*P*
Demographics						
Age, years	69.50(60.00,80.00)	69.00(59.00,80.00)	70.00(60.00,80.00)	70.00(60.00,80.00)	69.00(59.00,78.00)	0.391
Gender, n (%)						
Male	1188(55.51)	307(56.33)	311(56.34)	278(53.36)	292(55.94)	0.725
Female	952(44.49)	238(43.67)	241(43.66)	243(46.64)	230(44.06)	
Past medical history						
Diabetes, n(%)						
No	1356(63.36)	304(55.78)	401(72.64)	365(70.06)	286(54.79)	< 0.001
Yes	784(36.64)	241(44.22)	151(27.36)	156(29.94)	236(45.21)	
Myocardial infarct, n(%)						
No	1651(77.15)	431(79.08)	440(79.71)	400(76.78)	380(72.80)	0.031
Yes	489(22.85)	114(20.92)	112(20.29)	121(23.22)	142(27.20)	
Congestive heart failure, n(%)						
No	1864(87.10)	477(87.52)	495(89.67)	454(87.14)	438(83.91)	0.044
Yes	276(12.90)	68(12.48)	57(10.33)	67(12.86)	84(16.09)	
Chronic pulmonary disease, n(%)						
No	1796(83.93)	453(83.12)	474(85.87)	436(83.69)	433(82.95)	0.532
Yes	344(16.07)	92(16.88)	78(14.13)	85(16.31)	89(17.05)	
Dementia, n(%)						
No	2039(95.28)	521(95.60)	529(95.83)	491(94.24)	498(95.40)	0.623
Yes	101(4.72)	24(4.40)	23(4.17)	30(5.76)	24(4.60)	
Malignant cancer, n(%)						
No	2022(94.49)	515(94.50)	533(96.56)	485(93.09)	489(93.68)	0.067
Yes	118(5.51)	30(5.50)	19(3.44)	36(6.91)	33(6.32)	
Peripheral vascular disease, n(%)						
No	1924(89.91)	486(89.17)	498(90.22)	471(90.40)	469(89.85)	0.914
Yes	216(10.09)	59(10.83)	54(9.78)	50(9.60)	53(10.15)	
Cerebrovascular disease, n(%)						
No	845(39.49)	207(37.98)	178(32.25)	194(37.24)	266(50.96)	< 0.001
Yes	1295(60.51)	338(62.02)	374(67.75)	327(62.76)	256(49.04)	
Mild liver disease, n(%)						
No	2030(94.86)	527(96.70)	537(97.28)	496(95.20)	470(90.04)	< 0.001
Yes	110(5.14)	18(3.30)	15(2.72)	25(4.80)	52(9.96)	
Severe liver disease, n(%)						
No	2115(98.83)	543(99.63)	551(99.82)	519(99.62)	502(96.17)	< 0.001
Yes	25(1.17)	2(0.37)	1(0.18)	2(0.38)	20(3.83)	
Metastatic solid tumor, n(%)						
No	2094(97.85)	534(97.98)	544(98.55)	511(98.08)	505(96.74)	0.212
Yes	46(2.15)	11(2.02)	8(1.45)	10(1.92)	17(3.26)	
Paraplegia, n(%)						
No	1398(65.33)	374(68.62)	351(63.59)	316(60.65)	357(68.39)	0.014
Yes	742(34.67)	171(31.38)	201(36.41)	205(39.35)	165(31.61)	
Peptic ulcer disease, n(%)						
No	2113(98.74)	541(99.27)	546(98.91)	516(99.04)	510(97.70)	0.100
Yes	27(1.26)	4(0.73)	6(1.09)	5(0.96)	12(2.30)	
Rheumatic disease, n(%)						
No	2090(97.66)	538(98.72)	540(97.83)	505(96.93)	507(97.13)	0.204
Yes	50(2.34)	7(1.28)	12(2.17)	16(3.07)	15(2.87)	
Laboratory indicators						
RBC,10^12/L	4.18(3.76,4.59)	4.22(3.81,4.64)	4.26(3.86,4.63)	4.16(3.77,4.57)	4.06(3.60,4.53)	< 0.001
WBC,10^9/L	9.70(7.40,12.60)	8.40(6.70,10.80)	9.20(7.20,11.70)	10.30(8.20,12.90)	11.80(8.80,15.18)	< 0.001
Platelet,10^9/L	217.00(174.00,268.00)	218.00(179.00,265.00)	221.00(175.00,269.25)	215.00(174.00,268.00)	213.5(166.00,268.75)	0.441
Hemoglobin,g/dL	12.60(11.30,13.90)	12.60(11.20,13.90)	12.90(11.70,13.90)	12.60(11.30,14.00)	12.30(10.90,13.80)	< 0.001
Hematocrit,vol%	38.00(34.30,41.40)	38.20(34.10,41.70)	38.60(35.20,41.53)	37.70(34.60,41.30)	37.00(32.70,40.90)	< 0.001
MCH,pg	30.30(28.90,31.53)	30.00(28.50,31.20)	31.40(29.10,33.30)	30.40(29.20,31.80)	30.40(28.90,31.78)	< 0.001
MCHC,g/L	33.20(32.30,34.20)	33.00(32.10,34.10)	33.00(32.40,34.10)	33.50(32.50,34.30)	33.20(32.30,34.10)	0.002
MCV,fL	91.00(87.00,94.00)	90.00(87.00,94.00)	91.00(87.00,94.00)	91.00(87.00,95.00)	91.00(87.00,95.00)	0.003
RDW,%	13.50(12.90,14.40)	13.60(12.90,14.60)	13.50(12.90.14.30)	13.50(12.80.14.20)	13.60(12.90,14.60)	0.029
Anion gap,mmol/L	14.00(12.00,16.00)	13.00(12.00,15.00)	14.00(12.00,15.00)	14.00(12.00,16.00)	15.00(13.00,18.00)	< 0.001
Bicarbonate,mmol/L	23.00(21.00,26.00)	24.00(22.00,26.00)	24.00(22.00,26.00)	23.00(22.00,25.00)	22.00(20.00,24.00)	< 0.001
BUN,mg/dL	16.00(12.00,21.00)	15.00(12.00,21.00)	15.00(12.00,20.00)	16.00(12.00,21.00)	18.00(14.00,24.75)	< 0.001
Calcium,mg/dL	9.40(8.80,9.20)	8.80(8.40,9.20)	8.90(8.60,9.20)	8.90(8.40,9.20)	8.70(8.30,9.20)	< 0.001
Chloride,mEq/L	103.00(100.00,106.00)	104.00(101.00,107.00)	103.00(101.00,106.00)	103.00(100.00,106.00)	102.00(99.00,105.00)	< 0.001
Creatinine,mg/dL	0.90(0.70,1.10)	0.90(0.70,1.00)	0.90(0.70,1.00)	0.90(0.70,1.00)	1.00(0.80,1.20)	< 0.001
Glucose,mg/dL	128.00(104.00,167.25)	99.00(89.00,125.00)	112.00(102.00,127.00)	132.00(119.00,149.00)	183.00(154.00,258.25)	< 0.001
HbA1c,%	5.80(5.50,6.80)	6.20(5.70,7.90)	5.80(5.40,6.30)	5.70(5.40,6.20)	5.90(5.40,7.10)	< 0.001
Potassium,mmol/L	4.00(3.70,4.30)	4.00(3.70,4.30)	4.00(3.70,4.30)	3.90(3.60,4.30)	4.10(3.70,4.50)	0.011
Sodium,mmol/L	139.00(136.00,141.00)	140.00(137.00,142.00)	139.00(137.00,141.00)	139.00(136.00,141.00)	138.00(136.00,140.00)	< 0.001
eGFR,mL/min/1.73m^2^	80.21(64.49,101.48)	81.86(67.78,103.12)	84.03(67.70,102.88)	83.94(64.88,103.03)	73.93(56.56,90.25)	< 0.001
Disease Severity Score						
Charlson Comorbidity Index	5.00(3.00,7.00)	5.00(3.00,7.00)	5.00(3.00,6.00)	5.00(4.00,7.00)	5.00(4.00,7.00)	0.061
APS III score	33.00(25.00,43.00)	31.00(24.00,39.00)	30.00(24.00,39.00)	33.00(25.00,43.00)	39.00(30.00,49.00)	< 0.001
SAPS II score	30.00(23.00,37.00)	29.00(22.00,35.00)	29.00(23.00,35.00)	30.00(24.00,37.00)	33.00(26.00,40.00)	< 0.001
OASIS score	29.00(24.00,35.00)	28.00(24.00,33.00)	29.00(24.00,34.00)	29.00(25.00,35.00)	30.00(25.00,37.00)	< 0.001
GCS score	15.00(14.00,15.00)	15.00(15.00,15.00)	15.00(14.00,15.00)	15.00(14.00,15.00)	15.00(14.00,15.00)	0.112
Treatments						
Diuretics,n(%)	293(13.69)	61(11.19)	65(11.78)	82(15.74)	85(16.28)	0.024
β-blockers,n(%)	1040(48.60)	244(44.77)	280(50.72)	271(52.02)	245(46.93)	0.066
Calcium Channel Blockers,n(%)	555(25.93)	113(20.73)	160(28.99)	150(28.79)	132(25.29)	0.005
ACEI/ARB,n(%)	289(13.50)	72(13.21)	68(12.32)	78(14.97)	71(13.60)	0.644
Insulin,n(%)	1398(65.33)	371(68.07)	323(58.51)	319(61.23)	385(73.75)	< 0.001
Statins,n(%)	832(38.88)	245(44.95)	205(37.14)	204(39.16)	178(34.10)	0.003

RBC, red blood cell; WBC, white blood cell; MCH, mean corpuscular hemoglobin; MCHC, mean corpuscular hemoglobin concentration; MCV, mean corpuscular volume; RDW, red blood cell distribution width; BUN, blood urea nitrogen; HbA1c, hemoglobin A1c; eGFR, estimated glomerular filtration rate; APSIII, acute physiology score; SAPSII, simplified acute physiology score; OASIS, oxford acute severity of illness score; GCS, glasgow coma scale.

### Statistical analysis

Continuous variables that followed a normal distribution were presented as mean ± standard deviation (SD), and the Student t-test or ANOVA was applied to compare differences between groups. For continuous variables that did not follow a normal distribution, they were expressed as median (interquartile range), and the Mann-Whitney U test was used for comparisons between two groups, while the Kruskal-Wallis H test was applied for comparisons among more than two groups. Categorical variables were reported as counts and percentages, and differences between groups were assessed using the chi-square test or Fisher’s exact test, as appropriate.

Kaplan-Meier (KM) curves were plotted to assess the relationship between the SHR index and all-cause mortality. To assess multicollinearity among the variables, the variance inflation factor (VIF) was used; a VIF value of 5 or higher indicates multicollinearity. Univariate and multivariate Cox regression were utilized to assess the association between continuous or categorical SHR and all-cause mortality, and the results were reported as hazard ratios (HR) and 95% confidence intervals (CI). Model 1 was unadjusted, Model 2 was corrected for age and sex, and Model 3 was further corrected for diabetes, cerebrovascular disease, anion gap, bicarbonate, bun, calcium, chloride, creatinine. The lowest quartile of the SHR index was designated as the reference group for comparison in the analysis. To assess the robustness of the study results under the assumption of missing data, we conducted a complete-case analysis (CCA) as a sensitivity analysis, including only participants with complete data on all covariates. The same multivariate Cox regression model used in the primary analysis was applied to the complete-case dataset. However, due to the inability to obtain time-dependent covariate data, the study did not address the Cox proportional hazards assumptions.

The overall nonlinear relationship between SHR index as a continuous variable and all-cause mortality was assessed using restricted cubic spline (RCS). After determining the nonlinear association, a two-stage cox proportional risk model was further used to determine the inflection point.

In addition, subgroup analyses were performed according to sex, age, diabetes, myocardial infarction, congestive heart failure, cerebrovascular disease, and history of paraplegia.

Variables with a missing rate exceeding 20% were excluded from the analysis (see S1 Table for detailed missing rates). For variables with a missing rate below 20%, a multiple imputation method based on random forests was applied; this algorithm was implemented using the missForest package in R software (version 4.4.1). In the MIMIC-IV database, the patterns of missing values for laboratory and clinical variables are typically associated with observed patient characteristics rather than with the unobserved values themselves. Therefore, this study employed the Random Forest method for data imputation within the framework of Missing at Random. All statistical analyses were performed using SPSS software (version 27.0) and R software (version 4.4.1). The significance level was set at *P* < 0.05 for all tests.

## Results

### Baseline characteristics of the study population

A total of 2,140 patients with hypertension were selected from the MIMIC-IV database for analysis. The mean age of the participants was 69.5 years, with an age range from 60 to 80 years. Among the participants, 55.51% were male. Based on the calculated SHR index, all patients were categorized into four groups according to quartiles. The baseline characteristics and medication data of these groups are shown in Table 1. In terms of laboratory indicators, individuals in the highest SHR index group (Q4) exhibited a greater frequency of diabetes, myocardial infarction, congestive heart failure, mild liver disease and severe liver disease. Additionally, they exhibited higher levels of white blood cell (WBC), anion gap, blood urea nitrogen (BUN), creatinine, glucose and potassium, along with lower levels of red blood cell (RBC), hemoglobin, hematocrit, bicarbonate, calcium, chloride, sodium and eGFR compared with the other groups.

For disease severity scores, Acute Physiology Score III (APS III), Simplified Acute Physiology Score II (SAPS II), and Oxford Acute Severity of Illness Score (OASIS) were higher in the Q4 group than the remaining three groups, and had the longest hospitalization and ICU stay. Meanwhile, the Q4 group used diuretics and insulin significantly more than the other groups. The Q4 group exhibited significantly higher all-cause mortality at various time points: 30-day mortality (Q4:10.92%, Q3:8.06%, Q2:6.52%; Q1:4.40%, *P* < 0.001), 90-day mortality (Q4:14.37%, Q3:10.56%, Q2:8.70%; Q1:8.26%, *P* = 0.004), 180-day mortality (Q4:16.67%, Q3:13.44%, Q2:10.69%; Q1:10.28%, *P =* 0.006), and 365-day mortality (Q4:20.11%, Q3:16.31%, Q2:12.50%; Q1:12.66%, *P* < 0.001) (Table 2).

**Table 2:** Clinical course and outcomes.

Variables	Total (n = 2140)	Q1 (SHR ≤ 0.86, n = 545)	Q2 (0.86 < SHR ≤ 1.02, n = 552)	Q3 (1.02 < SHR ≤ 1.24, n = 521)	Q4 (SHR > 1.24, n = 522)	*P*
LOS						
LOS in hospital, days	6.72(3.93,11.79)	5.82(3.24,9.15)	5.96(3.68,10.03)	6.98(4.65,12.91)	8.58(4.91,15.18)	< 0.001
LOS in ICU, days	2.75(1.75,5.01)	2.18(1.59,3.93)	2.75(1.69,4.64)	2.86(1.82,5.43)	3.19(1.90,6.44)	< 0.001
Outcomes						
Hospital Death, n (%)						
No	2063(96.40)	537(98.53)	531(96.20)	502(96.35)	493(94.44)	0.005
Yes	77(3.60)	8(1.47)	21(3.80)	19(3.65)	29(5.56)	
30-day mortality, n (%)						
No	1981(92.57)	521(95.60)	516(93.48)	479(91.94)	465(89.08)	< 0.001
Yes	159(7.43)	24(4.40)	36(6.52)	42(8.06)	57(10.92)	
90-day mortality, n (%)						
No	1917(89.58)	500(91.74)	504(91.30)	466(89.44)	447(85.63)	0.004
Yes	223(10.42)	45(8.26)	48(8.70)	55(10.56)	75(14.37)	
180-day mortality, n (%)						
No	1868(87.29)	489(89.72)	493(89.31)	451(86.56)	435(83.33)	0.006
Yes	272(12.71)	56(10.28)	59(10.69)	70(13.44)	87(16.67)	
365-day mortality, n (%)						
No	1812(84.67)	476(87.34)	483(87.50)	436(83.69)	417(79.89)	0.001
Yes	328(15.33)	69(12.66)	69(12.50)	85(16.31)	105(20.11)	

LOS, Length Of Stay; ICU, Intensive Care Unit.

### Study outcomes

**Fig 2:**
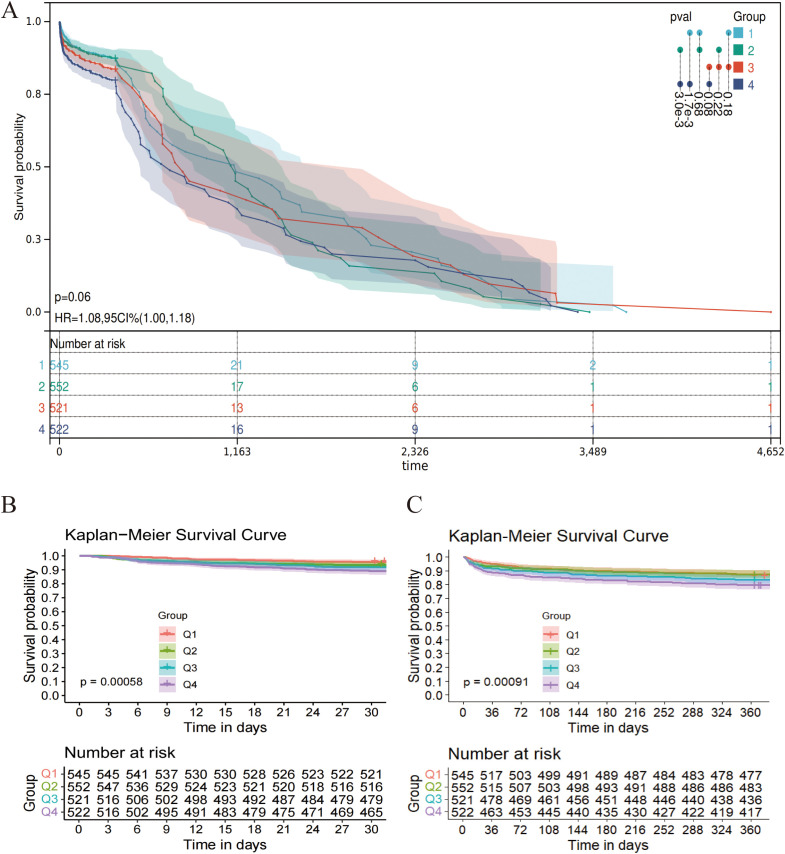
(A) KM curve for overall survival time, the x-axis represents survival time in days (B) KM curve for 30-day mortality (C) KM curve for 365-day mortality.

The KM curves (Fig 2) showed the survival curves for the four SHR quartile groups. We first performed a KM analysis of patient survival time, and the results of the study showed that the highest SHR index group (Q4) had a lower survival rate than the lower SHR index groups (Q1 and Q2). Further analysis of patient survival at 30, 90, 180 and 365 days revealed that there was a significant differences in mortality among the four SHR quartile groups. Patients in the highest SHR index group (Q4) had significantly lower survival rates at 30, 90, 180 and 365 days than those in the lower SHR index group (log-rank *P* < 0.05). Of these, the 90 and 180 days KM curves are shown in S1 Fig.

### Correlation of the SHR index with all-cause mortality in critically ill patients with hypertension

To evaluate the independent impact of the SHR index on all-cause mortality, we employed Cox regression analysis. Model 1 (unadjusted) shows that patients in the highest quartile (Q4) had a significantly higher risk of death than those in the lowest quartile (Q1) (Tables 3 and 4). After adjustment for age and sex (Model 2) the effect remained essentially unchanged. Model 3 further adjustment for diabetes, myocardial infarct, congestive heart failure, cerebrovascular disease, anion gap, bicarbonate, bun, calcium, chloride, creatinine, sodium and eGFR, with no significant multicollinearity detected (S2 Table). We found the same results. These findings indicate that higher SHR values are significantly associated with increased mortality risk, a trend also observed in models for 90- and 180-day mortality (S3 Table and S4 Table).

**Table 3:** Cox proportional hazard models for 30-day all-cause mortality.

Variables	Model 1	HR (95% CI)	Model 2	HR (95% CI)	Model 3	HR (95% CI)
	*P*		*P*		*P*	
SHR quantile						
1	1.00(Reference)		1.00(Reference)		1.00(Reference)	
2	1.51(0.90 ~ 2.53)	0.117	1.56(0.93 ~ 2.62)	0.090	1.55(0.92 ~ 2.62)	0.098
3	1.87(1.13 ~ 3.09)	0.014	1.91(1.15 ~ 3.15)	0.012	1.98(1.19 ~ 3.29)	0.009
4	2.57(1.59 ~ 4.14)	< 0.001	2.91(1.80 ~ 4.69)	< 0.001	2.79(1.70 ~ 4.59)	< 0.001
HR for trend	1.35(1.17 ~ 1.56)		1.40(1.21 ~ 1.62)		1.39(1.19 ~ 1.61)	
*P* for trend		< 0.001		< 0.001		< 0.001

Model 1: Crude.

Model 2: Adjust: Gender, Age.

Model 3: Adjust: Gender, Age, Diabetes, Cerebrovascular disease, Anion gap, Bicarbonate, Bun, Calcium, Chloride, Creatinine.

**Table 4:** Cox proportional hazard models for 365-day all-cause mortality.

Variables	Model 1	HR(95% CI)	Model 2	HR(95% CI)	Model 3	HR(95% CI)
	*P*		*P*		*P*	
SHR quantile						
1	1.00(Reference)		1.00(Reference)		1.00(Reference)	
2	1.00(0.72 ~ 1.40)	0.999	1.02(0.73 ~ 1.43)	0.898	1.00(0.72 ~ 1.40)	0.991
3	1.33(0.96 ~ 1.82)	0.082	1.34(0.98 ~ 1.84)	0.071	1.33(0.97 ~ 1.84)	0.079
4	1.68(1.24 ~ 2.27)	0.001	1.88(1.39 ~ 2.55)	< 0.001	1.77(1.29 ~ 2.43)	< 0.001
HR for trend	1.21(1.10 ~ 1.33)		1.25(1.13 ~ 1.38)		1.23(1.11 ~ 1.36)	
*P* for trend		< 0.001		< 0.001		< 0.001

Model 1: Crude.

Model 2: Adjust: Gender, Age.

Model 3: Adjust: Gender, Age, Diabetes, Cerebrovascular disease, Anion gap, Bicarbonate, Bun, Calcium, Chloride, Creatinine.

To verify the robustness of the main analysis results, we conducted a CCA study. Among the 2,140 participants included in the analysis, 1,817 (84.91%) had complete data for all covariates and were therefore included in the CCA. As shown in S5–S8 Tables, the HRs for SHR in the CCA were similar in direction and magnitude to those obtained from the primary multiple imputation analysis, indicating that missing data did not substantially bias our primary conclusions.

### Nonlinear relationship between SHR index and mortality in critically ill patients with hypertension

**Fig 3:**
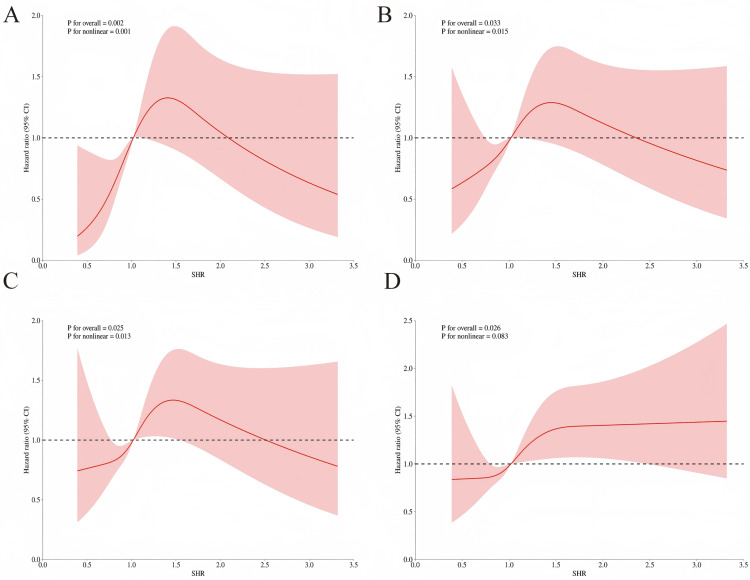
RCS of SHR index with all-cause mortality at 30 days (A), 90 days (B), 180 days (C), and 365 days (D).

RCS curves revealed a significant inverted U-shaped (nonlinear) association between SHR and all-cause mortality (*P* < 0.05 at 30, 90, and 180 days, but *P* > 0.05 at 365 days) (see Fig 3). To identify the cutoff point, we employed a two-stage Cox regression approach, searching for the cutoff value that maximized the log-likelihood ratio statistic (i.e., minimized the *P*-value of the likelihood ratio test) across a range of candidate SHR values. The results showed that SHR = 1.76 was the optimal cutoff value for 30-day, 90-day, and 180-day all-cause mortality (likelihood ratio test *P* < 0.001) (see Table 5, S9 Table and S10 Table). Specifically, when the SHR value is below 1.76, the risk of death increases as the SHR rises; when the SHR value is above 1.76, the risk appears to level off or decrease slightly. However, since this threshold is derived from the current dataset, it should be considered an exploratory finding and requires external validation prior to clinical application.

**Table 5:** Threshold effect analysis of SHR index on 30-day all-cause mortality in patients with hypertension.

30-day mortality	HR (95% CI)	*P*-value
Model I Fitting Model by standard linear regression	1.37 (1.04, 1.82)	0.027
Model II Fitting Model by two-piecewise linear regression		
Inflection point	1.76	
SHR < 1.76	3.87 (2.22, 6.75)	< 0.001
SHR > 1.76	0.39 (0.14, 1.13)	0.083
*P* for Log-likelihood ratio		< 0.001

### Subgroup analysis

**Fig 4:**
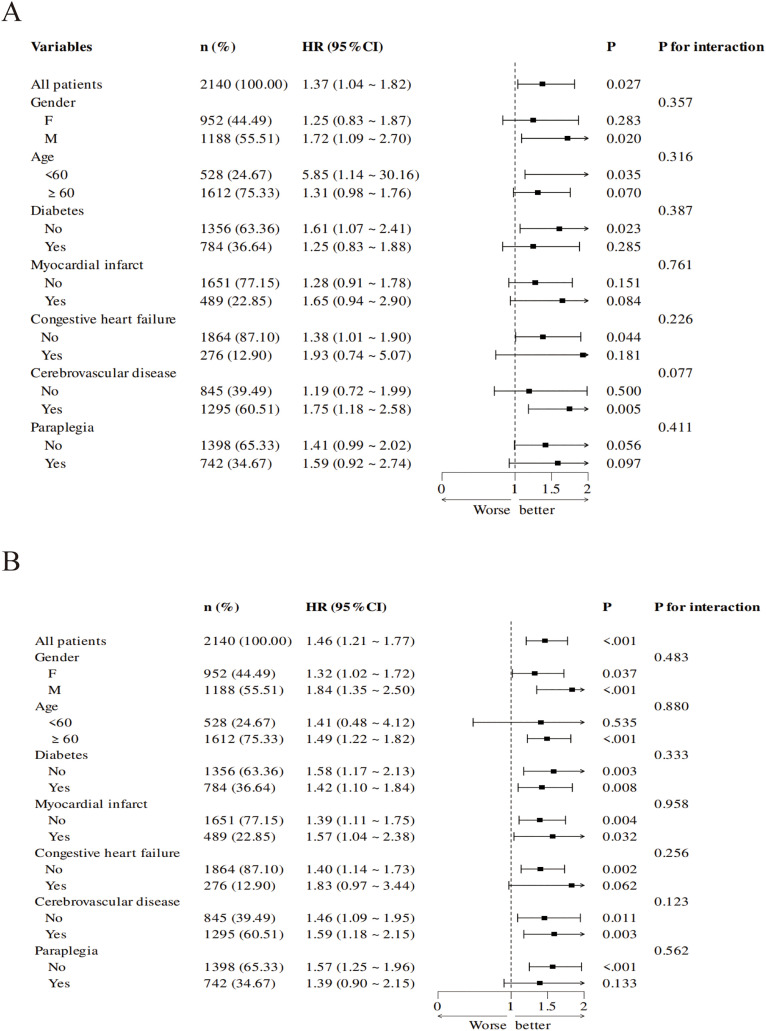
Forest plots of 30-day (A) and 365-day (B) all-cause mortality rates.

Subgroup analyses were conducted for gender, age, diabetes, myocardial infarction, congestive heart failure, cerebrovascular disease, and paraplegia to explore the consistency of the association between SHR levels and all-cause mortality across different conditions and time points. For 30-day all-cause mortality, significant differences were observed in men and across age groups, as well as in patients with diabetes and congestive heart failure. At 90 days, significant differences were noted in older patients, those with diabetes, and those with cerebrovascular disease. By 180 days, gender, age, diabetes, and paraplegia subgroups showed significant differences. At the 365-day mark, all subgroups exhibited significant differences in all-cause mortality (Fig 4, S2 Fig). Notably, the findings indicate that the association between SHR and mortality is more pronounced in non-diabetic individuals.

In the gender subgroup, men experienced significantly higher all-cause mortality rates at 30 and 180 days (*P* < 0.05). SHR levels were significantly associated with 30-day mortality in patients under 60 years old. In patients over 60, significant HRs were observed for 90-, 180-, and 365-day mortality (*P* < 0.05). Notably, the HR for all-cause mortality was significant both in the short and long term for patients without congestive heart failure (*P* < 0.05) (Fig 4). Furthermore, HRs for 180 and 365-day mortality were significant in patients with uncomplicated paraplegia (Fig 4B, S2 Fig). SHR levels significantly correlated with 365-day all-cause mortality regardless of the presence or absence of myocardial infarction or cerebrovascular disease (*P* < 0.05). And the association between SHR and all-cause mortality was consistent across all subgroups, with no significant interactions detected (all *P* for interaction > 0.05) (Fig 4, S2 Fig).

## Discussion

Our study was the first to investigate the relationship between SHR levels and all-cause mortality in critically ill patients with hypertension and showed that mortality in critically ill patients with hypertension increased with increasing SHR index. At 30, 90 and 180 days, SHR showed an inverse U-shaped correlation with all-cause mortality, characterized by an inflection point at 1.76. The findings of this study may provide guidance for future treatment of critically ill patients with hypertension, with the potential to further reduce the risk of mortality. It is important to emphasize that SHR should be understood as a prognostic marker rather than an independent causal risk factor.

### The pathogenesis of increased all-cause mortality due to SHR

Cox regression analysis indicated that patient mortality increased as the SHR index rose, and RCS analysis revealed a significant nonlinear relationship between the SHR index and all-cause mortality at 30, 90 and 180 days. The risk of death was highest when the SHR inflection point was 1.76. The results align with prior research indicating that elevated SHR values are linked to higher risks of in-hospital and all-cause mortality [12,36], suggesting that SHR can serve as an potential predictor of mortality in critically ill patients [8]. However, since no formal proportional hazards test was conducted, these associations should be considered exploratory. Nevertheless, confirmatory studies incorporating rigorous proportional hazards tests are still needed before clinical application.

The mechanisms by which elevated SHR leads to increased mortality are unclear, and it has been shown that SIH states intensify the inflammatory cascade, elevate oxidative stress, compromise vascular function, and promote cytokine release, which leads to adverse clinical outcomes [37,38]. Stress hyperglycemia has been shown to amplify the inflammatory immune response and is linked to higher mortality rates in patients with myocardial infarction [39]. At the same time, SIH can trigger oxidative stress, leading to hyperactivation of reactive oxygen species (ROS) [40] and promoting the emergence of platelet activation and aggregation [41]. Hyperglycemia also damages vascular endothelial cells through complex mechanisms, including activation of protein kinase C, leukocyte adhesion and uptake, endothelial cell proliferation, synthesis of collagen IV and fibronectin, and reduced production of atheroprotective nitric oxide (NO) [42]. Uncontrolled glucose levels in hospitalized patients are associated with potential harms, such as adverse effects on wound healing, increased risk of infection, ultimately leading to non-hyperglycemic death [43].

For hypertensive patients, SHR or hyperglycemia can significantly increase mortality risk through multiple interrelated pathophysiological mechanisms. First, SHR enhances sympathetic activity, promoting increased catecholamine release, elevating peripheral vascular resistance, and ultimately leading to hypertension and increased cardiovascular workload [44]. Concurrently, hyperglycemia directly or indirectly activates the sympathetic nervous system through the release of inflammatory mediators, creating a vicious cycle [45,46]. Second, SHR is closely associated with oxidative stress and inflammatory responses. Chronic hyperglycemia promotes excessive ROS production, damaging vascular endothelial cells and reducing NO bioavailability, thereby impairing endothelium-dependent vasodilation [47,48]. Moreover, oxidative stress induces low-grade chronic inflammation in the vascular wall, further impairing endothelial function and promoting arterial stiffness [49]. Under conditions of insulin resistance or hyperinsulinemia, the Renin-Angiotensin-Aldosterone System (RAAS) is also overactivated, subsequently causing vasoconstriction, sodium and water retention, and vascular remodeling, thereby contributing to the development and progression of hypertension and its complications [44,50,51]. In summary, SHR synergistically promotes vascular endothelial dysfunction and arterial stiffness through multiple mechanisms, including sympathetic hyperactivity, RAAS activation, oxidative stress, and inflammatory responses, thereby exacerbating hypertension and increasing patient mortality.

Although elevated SHR values are typically associated with increased mortality, the observed risk reduction beyond the data‑driven cutoff (SHR = 1.76) warrants cautious interpretation. First, this threshold was identified using an optimization procedure on the current dataset; therefore, it may be overfitted to sample‑specific characteristics and might not generalize directly to other populations. Second, extremely high SHR values may trigger compensatory mechanisms, such as enhanced insulin sensitivity or activation of protective pathways, thereby mitigating the adverse effects of hyperglycemia [52]. Third, such patients may receive more intensive medical interventions, thereby improving their prognosis [23]. Given the exploratory nature of the threshold, its clinical relevance should be evaluated in independent external cohorts. Until such validation is available, the identified cutoff (1.76) should not be used for individual risk stratification or treatment decisions.

### Prognosis of patients with high SHR and differences between subgroups

Subgroup analyses were exploratory in nature and were not adjusted for multiple comparisons; therefore, the findings should be interpreted as hypothesis‑generating rather than confirmatory. Despite this limitation, our subgroup analysis suggests that the association between SHR and mortality may vary across different gender and age groups, factors that are critical in assessing cardiovascular risk in patients with hypertension. Previous studies have also demonstrated combining the SHR index with traditional risk factors improves the prediction of in-hospital and all-cause mortality in ST-segment Elevation Myocardial Infarction (STEMI) patients [36], supporting the general concept that SHR could serve as a supplementary tool to existing risk assessment strategies. Similarly, SHR has been considered a potential predictor of cerebrovascular disease [24,53,54], a condition closely associated with hypertension. The underlying mechanisms include ROS production leading to apoptosis of vascular endothelial cells, which exacerbates cerebral perfusion disorders and neurological deficits-a scenario particularly relevant in the context of hypertension. Counter-regulatory hormones are increased in critical illnesses, releasing inflammatory cytokines that antagonize insulin or impair insulin signaling and exacerbate hyperglycemia [23]. The interaction between hyperglycemia and hypertension is critical, as it may lead to a vicious cycle that further worsens cardiovascular outcomes. Given the exploratory nature and the lack of multiplicity adjustment, these subgroup findings require confirmation in independent prospective cohorts before any firm conclusions can be drawn.

Interestingly, subgroup analyses showed that non-diabetic patients with high SHR exhibited higher all-cause mortality than patients with diabetes, consistent with the findings of several studies [55–57]. For example, Chen et al. subgroup analysis showed that SHR values above 1.25 were significantly associated with in-hospital mortality in elderly acute myocardial infarction nondiabetic patients, even after adjusting for potential confounders, but not in patients with diabetes [56]. The same results were shown in critically ill patients with acute kidney injury, where the observed association between higher SHR and ICU mortality was only significant in non-diabetic patients [57]. This is consistent with the concept of the “diabetes paradox” in the ICU, where diabetes is not independently associated with an increased risk of death in critically ill patients [58]. The current possible explanation is that diabetes itself leads to a poor long-term prognosis and therefore the effect of high SHR is masked in diabetic patients [59]. On the other hand, the inflammatory response is more prominent in newly diagnosed hyperglycemic patients than in those with known diabetes [39]. And hyperglycemic patients without known diabetes are less likely to receive insulin therapy [59]. Meanwhile, subgroup analysis of interaction results revealed that different subgroup characteristics did not significantly alter the strength of the association between SHR and mortality, indicating the robustness of SHR’s prognostic value.

### Significance and shortcomings of this study

Our study suggests that the SHR index could serve as a potential tool for clinically assessing critically ill patients with hypertension. As an easily accessible and calculated index, the SHR index may help physicians to quickly assess the prognosis of patients and adopt appropriate treatment to reduce mortality.

The novelty of this study lies in establishing that SHR serves as a prognostic marker associated with mortality in patients with hypertension, rather than an independent causal risk factor. Given the high prevalence of hypertension, this patient cohort constitutes a large population within ICUs, yet its specific risk profile is often overlooked. Previous studies lacked dedicated investigation into the association between SHR and outcomes in this group, a gap addressed by this research. These findings help generate new hypotheses and suggest that future research could evaluate whether incorporating SHR into standardized assessment protocols for patients with hypertension would aid in identifying high-risk individuals. For patients with elevated SHR values, the frequency of monitoring frequency of blood pressure, blood glucose levels, and other vital signs should be intensified to detect early signs of clinical deterioration. When SHR indicates higher risk, more aggressive management strategies should be considered, such as stricter blood glucose control and intensified antihypertensive therapy.

This study has several limitations. First, the MIMIC-IV database utilized for the study was a retrospective observational database. Although we performed rigorous multivariate adjustments for covariates, residual confounding factors, particularly those arising from unmeasured and time-dependent variables, such as blood glucose fluctuations and therapeutic interventions, may have influenced the observed association between SHR and mortality. Second, the timing of the first glucose measurement (fasting vs. random) may introduce variability and impact study results; however, this specific data cannot be obtained from the MIMIC-IV database. Therefore, we used the glycated hemoglobin value in the SHR formula to mitigate this variability. And it is important to note that the MIMIC-IV database now includes data from 2020–2022, which encompasses the Coronavirus Disease (COVID) pandemic period and may have influenced patient management and outcomes. Therefore, the observed association between SHR and mortality may be subject to bias related to the pandemic context. Future studies should consider the unique impact of the pandemic on patient outcomes and medical practice. In addition, the study primarily focused on elderly and Caucasian populations, limiting the generalizability of the findings. Furthermore, a major limitation is that the proportional hazards assumption was not formally tested due to database constraints. Violation could make our hazard ratios time‑averaged rather than constant, potentially biasing interpretation. Thus, our conclusions are exploratory and require validation in future studies with formal PH testing. Finally, this study was unable to distinguish between patients with type 1 and type 2 diabetes, which may affect glycemic control and consequently influence SHR. The absence of such data may introduce bias or confounding factors, limiting our conclusions regarding the independent impact of SHR on mortality. Future studies should consider incorporating detailed data to better understand the relationship between SHR, medication use, and mortality.

## Conclusion

The SHR index is associated with prognosis in critically ill patients with hypertension in the ICU, suggesting its potential utility for risk stratification. However, this association does not imply causality, and its validity and generalizability need to be confirmed by further prospective studies.

## Supporting information

S1 Table Proportion of missing data for all variables. (DOCX)

S2 Table Results of the multicollinearity diagnosis for the variables in the Cox regression model 3. (DOCX)

S3 Table Cox proportional hazard models for 90-day all-cause mortality. (DOCX)

S4 Table Cox proportional hazard models for 180-day all-cause mortality. (DOCX)

S5 Table Cox proportional hazard models for 30-day all-cause mortality (complete case analysis). (DOCX)

S6 Table Cox proportional hazard models for 90-day all-cause mortality (complete case analysis). (DOCX)

S7 Table Cox proportional hazard models for 180-day all-cause mortality (complete case analysis). (DOCX)

S8 Table Cox proportional hazard models for 365-day all-cause mortality (complete case analysis). (DOCX)

S9 Table Threshold effect analysis of SHR index on 90-day all-cause mortality in patients with hypertension. (DOCX)

S10 Table Threshold effect analysis of SHR index on 180-day all-cause mortality in patients with hypertension. (DOCX)

S1 Fig Kaplan-Meier curve for 90-day (A) and 180-day (B) all-cause mortality. (TIF)

S2 Fig Forest plots of stratified analyses of SHR index with 90-day (A) and 180-day (B) all-cause mortality. (TIF)
