# Supplementary material for: The prognostic significance of stress hyperglycemic ratio in critically Ill patients with hypertension: A study using the MIMIC-IV database
Source: PLoS One. 2026 Jul 31;21(7):e0352162. doi: 10.1371/journal.pone.0352162 (PMC13426943; doi:10.1371/journal.pone.0352162)
Supplement: S1 Table — (DOCX) [file pone.0352162.s001.docx]

**S1 Table. Proportion of missing data for all variables.**

| Variable name | Missing number（n,%） | |
| --- | --- | --- |
| rdwsd | 2140 (100) | |
| nrbc | 2126 (99.35) | |
| atypical_lymphocytes | 2095 (97.90) | |
| metamyelocytes | 2091 (97.71) | |
| bands | 2086 (97.48) | |
| globulin | 2004 (93.64) | |
| total_protein | 1998 (93.36) | |
| immature_granulocytes | 1793 (83.79) | |
| mbp | 1731 (80.89) | |
| sbp | 1720 (80.37) | |
| dbp | 1719 (80.33) | |
| temperature | 1575 (73.60) | |
| basophils | 1536 (71.78) | |
| basophils_abs | 1536 (71.78) | |
| eosinophils | 1536 (71.78) | |
| eosinophils_abs | 1536 (71.78) | |
| lymphocytes | 1536 (71.78) | |
| lymphocytes_abs | 1536 (71.78) | |
| monocytes | 1536 (71.78) | |
| monocytes_abs | 1536 (71.78) | |
| neutrophils | 1536 (71.78) | |
| neutrophils_abs | 1536 (71.78) | |
| albumin | 1344 (62.80) | |
| spo2 | 1284 (60.00) | |
| height | 1232 (57.57) | |
| heart_rate | 1073 (50.14) | |
| resp_rate | 900 (42.06) | |
| Calcium | 318 (14.86) |  |
| MCH | 16 (0.75) |  |
| MCHC | 16 (0.75) |  |
| RBC | 15 (0.70) |  |
| Hemoglobin | 15 (0.70) |  |
| MCV | 15 (0.70) |  |
| RDW | 15 (0.70) |  |
| WBC | 13 (0.61) |  |
| Platelet | 12 (0.56) |  |
| Aniongap | 12 (0.56) |  |
| Bicarbonate | 12 (0.56) |  |
| BUN | 8 (0.37) |  |
| Creatinine | 8 (0.37) |  |
| Hematocrit | 7 (0.33) |  |
| Chloride | 7 (0.33) |  |
| Potassium | 7 (0.33) |  |
| Sodium | 6 (0.28) |  |
| Glucose | 0 (0.00) |  |
| HbA1c | 0 (0.00) |  |
| Age | 0 (0.00) |  |
| Gender | 0 (0.00) |  |
| Diabetes | 0 (0.00) |  |
| Myocardial infarct | 0 (0.00) |  |
| Congestive heart failure | 0 (0.00) |  |
| Chronic pulmonary disease | 0 (0.00) |  |
| Dementia | 0 (0.00) |  |
| Malignant cancer | 0 (0.00) |  |
| Peripheral vascular disease | 0 (0.00) |  |
| Cerebrovascular disease | 0 (0.00) |  |
| Mild liver disease | 0 (0.00) |  |
| Severe liver disease | 0 (0.00) |  |
| Metastatic solid tumor | 0 (0.00) |  |
| Paraplegia | 0 (0.00) |  |
| Peptic ulcer disease | 0 (0.00) |  |
| Rheumatic disease | 0 (0.00) |  |
| APS III score | 0 (0.00) |  |
| SAPS II score | 0 (0.00) |  |
| OASIS score | 0 (0.00) |  |
| GCS score | 0 (0.00) |  |
| Diuretics | 0 (0.00) |  |
| β-blockers | 0 (0.00) |  |
| Calcium Channel Blockers | 0 (0.00) |  |
| ACEI/ARB | 0 (0.00) |  |
| Insulin | 0 (0.00) |  |
| Statins | 0 (0.00) |  |
