# Supplementary material for: The prognostic significance of stress hyperglycemic ratio in critically Ill patients with hypertension: A study using the MIMIC-IV database
Source: PLoS One. 2026 Jul 31;21(7):e0352162. doi: 10.1371/journal.pone.0352162 (PMC13426943; doi:10.1371/journal.pone.0352162)
Supplement: S2 Table — (DOCX) [file pone.0352162.s002.docx]

**S2 Table. Results of the multicollinearity diagnosis for the variables in the Cox regression model 3.**

| Constant | VIF |
| --- | --- |
| gender | 1.095 |
| age | 1.162 |
| diabetes | 1.069 |
| cerebrovascular_disease | 1.118 |
| aniongap | 1.922 |
| bicarbonate | 1.799 |
| bun | 2.015 |
| calcium | 1.162 |
| chloride | 1.353 |
| creatinine | 1.968 |
