# Supplementary material for: The prognostic significance of stress hyperglycemic ratio in critically Ill patients with hypertension: A study using the MIMIC-IV database
Source: PLoS One. 2026 Jul 31;21(7):e0352162. doi: 10.1371/journal.pone.0352162 (PMC13426943; doi:10.1371/journal.pone.0352162)
Supplement: S3 Table — (DOCX) [file pone.0352162.s003.docx]

**S3 Table. Cox proportional hazard models for 90-day all-cause mortality.**

| Variables | Model 1 |  | Model 2 |  | Model 3 |  |
| --- | --- | --- | --- | --- | --- | --- |
|  | HR(95% CI) | *P* | HR(95% CI) | *P* | HR(95% CI) | *P* |
| SHR quantile |  |  |  |  |  |  |
| 1 | 1.00(Reference) |  | 1.00(Reference) |  | 1.00(Reference) |  |
| 2 | 1.07(0.71~1.61) | 0.736 | 1.10(0.73~1.65) | 0.646 | 1.09(0.72~1.64) | 0.690 |
| 3 | 1.31(0.88~1.94) | 0.179 | 1.32(0.89~1.96) | 0.163 | 1.35(0.91~2.02) | 0.138 |
| 4 | 1.82(1.26~2.63) | 0.002 | 2.04(1.41~2.95) | < 0.001 | 1.92(1.31~2.82) | 0.001 |
| HR for trend | 1.23(1.09~1.39) |  | 1.28(1.13~1.44) |  | 1.25(1.10~1.42) |  |
| *P* for trend |  | 0.001 |  | < 0.001 |  | < 0.001 |

HR: Hazard Ratio, CI: Confidence Interval

Model 1: Crude

Model 2: Adjust: Gender, Age

Model 3: Adjust: Gender, Age，Diabetes, Cerebrovascular disease, Aniongap, Bicarbonate, Bun, Calcium, Chloride, Creatinine
