# Supplementary material for: The prognostic significance of stress hyperglycemic ratio in critically Ill patients with hypertension: A study using the MIMIC-IV database
Source: PLoS One. 2026 Jul 31;21(7):e0352162. doi: 10.1371/journal.pone.0352162 (PMC13426943; doi:10.1371/journal.pone.0352162)
Supplement: S4 Table — (DOCX) [file pone.0352162.s004.docx]

**S4 Table. Cox proportional hazard models for 180-day all-cause mortality.**

| Variables | Model 1 |  | Model 2 |  | Model 3 |  |
| --- | --- | --- | --- | --- | --- | --- |
|  | HR(95% CI) | *P* | HR(95% CI) | *P* | HR(95% CI) | *P* |
| SHR quantile |  |  |  |  |  |  |
| 1 | 1.00(Reference) |  | 1.00(Reference) |  | 1.00(Reference) |  |
| 2 | 1.06(0.73~1.52) | 0.772 | 1.08(0.75~1.56) | 0.683 | 1.05(0.72~1.51) | 0.809 |
| 3 | 1.34(0.94~1.91) | 0.102 | 1.35(0.95~1.92) | 0.092 | 1.36(0.95~1.94) | 0.091 |
| 4 | 1.70(1.22~2.38) | 0.002 | 1.91(1.36~2.67) | < 0.001 | 1.85(1.31~2.63) | 0.001 |
| HR for trend | 1.21(1.09~1.35) |  | 1.25(1.12~1.40) |  | 1.24(1.11~1.39) |  |
| *P* for trend |  | 0.001 |  | < 0.001 |  | < 0.001 |

HR: Hazard Ratio, CI: Confidence Interval

Model 1: Crude

Model 2: Adjust: Gender, Age

Model 3: Adjust: Gender, Age，Diabetes, Cerebrovascular disease, Aniongap, Bicarbonate, Bun, Calcium, Chloride, Creatinine
