# Supplementary material for: The prognostic significance of stress hyperglycemic ratio in critically Ill patients with hypertension: A study using the MIMIC-IV database
Source: PLoS One. 2026 Jul 31;21(7):e0352162. doi: 10.1371/journal.pone.0352162 (PMC13426943; doi:10.1371/journal.pone.0352162)
Supplement: S5 Table — (DOCX) [file pone.0352162.s005.docx]

**S5 Table. Cox proportional hazard models for 30-day all-cause mortality (complete case analysis).**

| Variables | Model 1 |  | Model 2 |  | Model 3 |  |
| --- | --- | --- | --- | --- | --- | --- |
|  | HR(95% CI) | *P* | HR(95% CI) | *P* | HR(95% CI) | *P* |
| SHR quantile |  |  |  |  |  |  |
| 1 | 1.00(Reference) |  | 1.00(Reference) |  | 1.00(Reference) |  |
| 2 | 1.64(0.95~2.85) | 0.079 | 1.69(0.97~2.94) | 0.063 | 1.71(0.98~2.98) | 0.059 |
| 3 | 1.75(1.01~3.03) | 0.046 | 1.78(1.03~3.09) | 0.039 | 1.88(1.08~3.28) | 0.027 |
| 4 | 2.34(1.39~3.95) | 0.001 | 2.53(1.50~4.28) | 0.001 | 2.51(1.46~4.33) | 0.001 |
| HR for trend | 1.28(1.10~1.50) |  | 1.31(1.13~1.54) |  | 1.31(1.12~1.55) |  |
| *P* for trend |  | 0.002 |  | 0.001 |  | 0.001 |

HR: Hazard Ratio, CI: Confidence Interval

Model 1: Crude

Model 2: Adjust: Gender, Age

Model 3: Adjust: Gender, Age，Diabetes, Cerebrovascular disease, Aniongap, Bicarbonate, Bun, Calcium, Chloride, Creatinine
