# Supplementary material for: The prognostic significance of stress hyperglycemic ratio in critically Ill patients with hypertension: A study using the MIMIC-IV database
Source: PLoS One. 2026 Jul 31;21(7):e0352162. doi: 10.1371/journal.pone.0352162 (PMC13426943; doi:10.1371/journal.pone.0352162)
Supplement: S6 Table — (DOCX) [file pone.0352162.s006.docx]

**S6 Table. Cox proportional hazard models for 90-day all-cause mortality (complete case analysis).**

| Variables | Model 1 |  | Model 2 |  | Model 3 |  |
| --- | --- | --- | --- | --- | --- | --- |
|  | HR(95% CI) | *P* | HR(95% CI) | *P* | HR(95% CI) | *P* |
| SHR quantile |  |  |  |  |  |  |
| 1 | 1.00(Reference) |  | 1.00(Reference) |  | 1.00(Reference) |  |
| 2 | 1.20(0.78~1.85) | 0.411 | 1.23(0.80~1.90) | 0.348 | 1.22(0.79~1.88) | 0.380 |
| 3 | 1.27(0.83~1.96) | 0.273 | 1.28(0.83~1.97) | 0.259 | 1.31(0.85~2.02) | 0.229 |
| 4 | 1.74(1.16~2.61) | 0.007 | 1.86(1.24~2.79) | 0.003 | 1.77(1.16~2.70) | 0.008 |
| HR for trend | 1.19(1.05~1.36) |  | 1.22(1.07~1.38) |  | 1.20(1.05~1.37) |  |
| *P* for trend |  | 0.007 |  | 0.003 |  | 0.008 |

HR: Hazard Ratio, CI: Confidence Interval

Model 1: Crude

Model 2: Adjust: Gender, Age

Model 3: Adjust: Gender, Age，Diabetes, Cerebrovascular disease, Aniongap, Bicarbonate, Bun, Calcium, Chloride, Creatinine
