# Supplementary material for: The prognostic significance of stress hyperglycemic ratio in critically Ill patients with hypertension: A study using the MIMIC-IV database
Source: PLoS One. 2026 Jul 31;21(7):e0352162. doi: 10.1371/journal.pone.0352162 (PMC13426943; doi:10.1371/journal.pone.0352162)
Supplement: S7 Table — (DOCX) [file pone.0352162.s007.docx]

**S7 Table. Cox proportional hazard models for 180-day all-cause mortality (complete case analysis).**

| Variables | Model 1 |  | Model 2 |  | Model 3 |  |
| --- | --- | --- | --- | --- | --- | --- |
|  | HR(95% CI) | *P* | HR(95% CI) | *P* | HR(95% CI) | *P* |
| SHR quantile |  |  |  |  |  |  |
| 1 | 1.00(Reference) |  | 1.00(Reference) |  | 1.00(Reference) |  |
| 2 | 1.22(0.83~1.80) | 0.321 | 1.25(0.84~1.84) | 0.269 | 1.21(0.82~1.80) | 0.339 |
| 3 | 1.31(0.89~1.94) | 0.169 | 1.32(0.89~1.94) | 0.164 | 1.33(0.90~1.97) | 0.154 |
| 4 | 1.71(1.18~2.47) | 0.004 | 1.83(1.26~2.64) | 0.001 | 1.82(1.24~2.67) | 0.002 |
| HR for trend | 1.19(1.06~1.33) |  | 1.21(1.08~1.36) |  | 1.21(1.07~1.37) |  |
| *P* for trend |  | 0.004 |  | 0.001 |  | 0.002 |

HR: Hazard Ratio, CI: Confidence Interval

Model 1: Crude

Model 2: Adjust: Gender, Age

Model 3: Adjust: Gender, Age，Diabetes, Cerebrovascular disease, Aniongap, Bicarbonate, Bun, Calcium, Chloride, Creatinine
