# Supplementary material for: The prognostic significance of stress hyperglycemic ratio in critically Ill patients with hypertension: A study using the MIMIC-IV database
Source: PLoS One. 2026 Jul 31;21(7):e0352162. doi: 10.1371/journal.pone.0352162 (PMC13426943; doi:10.1371/journal.pone.0352162)
Supplement: S8 Table — (DOCX) [file pone.0352162.s008.docx]

**S8 Table. Cox proportional hazard models for 365-day all-cause mortality (complete case analysis).**

| Variables | Model 1 |  | Model 2 |  | Model 3 |  |
| --- | --- | --- | --- | --- | --- | --- |
|  | HR(95% CI) | *P* | HR(95% CI) | *P* | HR(95% CI) | *P* |
| SHR quantile |  |  |  |  |  |  |
| 1 | 1.00(Reference) |  | 1.00(Reference) |  | 1.00(Reference) |  |
| 2 | 1.14(0.79~1.63) | 0.482 | 1.16(0.81~1.66) | 0.412 | 1.14(0.80~1.64) | 0.470 |
| 3 | 1.35(0.95~1.92) | 0.090 | 1.36(0.96~1.93) | 0.086 | 1.36(0.96~1.93) | 0.087 |
| 4 | 1.74(1.25~2.43) | 0.001 | 1.87(1.34~2.62) | < 0.001 | 1.83(1.29~2.58) | 0.001 |
| HR for trend | 1.21(1.09~1.34) |  | 1.23(1.11~1.37) |  | 1.23(1.10~1.37) |  |
| *P* for trend |  | < 0.001 |  | < 0.001 |  | < 0.001 |

HR: Hazard Ratio, CI: Confidence Interval

Model 1: Crude

Model 2: Adjust: Gender, Age

Model 3: Adjust: Gender, Age，Diabetes, Cerebrovascular disease, Aniongap, Bicarbonate, Bun, Calcium, Chloride, Creatinine
