# Supplementary material for: The prognostic significance of stress hyperglycemic ratio in critically Ill patients with hypertension: A study using the MIMIC-IV database
Source: PLoS One. 2026 Jul 31;21(7):e0352162. doi: 10.1371/journal.pone.0352162 (PMC13426943; doi:10.1371/journal.pone.0352162)
Supplement: S9 Table — (DOCX) [file pone.0352162.s009.docx]

**S9 Table. Threshold effect analysis of SHR index on 90-day all-cause mortality in patients with hypertension.**

| 90-day mortality | HR (95% CI) | *P*-value |
| --- | --- | --- |
| Model I Fitting Model by standard linear regression | 1.27 (1.00, 1.62) | 0.046 |
| Model II Fitting Model by two-piecewise linear regression |  |  |
| Inflection point | 1.76 |  |
| SHR < 1.76 | 2.63 (1.66, 4.18) | < 0.001 |
| SHR > 1.76 | 0.65 (0.35, 1.18) | 0.155 |
| *P* for Log-likelihood ratio |  | < 0.001 |
