# Supplementary material for: The prognostic significance of stress hyperglycemic ratio in critically Ill patients with hypertension: A study using the MIMIC-IV database
Source: PLoS One. 2026 Jul 31;21(7):e0352162. doi: 10.1371/journal.pone.0352162 (PMC13426943; doi:10.1371/journal.pone.0352162)
Supplement: S10 Table — (DOCX) [file pone.0352162.s010.docx]

**S10 Table. Threshold effect analysis of SHR index on 180-day all-cause mortality in patients with hypertension.**

| 180-day mortality | HR (95% CI) | *P*-value |
| --- | --- | --- |
| Model I Fitting Model by standard linear regression | 1.32 (1.05, 1.66) | 0.017 |
| Model II Fitting Model by two-piecewise linear regression |  |  |
| Inflection point | 1.76 |  |
| SHR < 1.76 | 2.62 (1.71, 4.01) | < 0.001 |
| SHR > 1.76 | 0.63 (0.34, 1.18) | 0.149 |
| *P* for Log-likelihood ratio |  | < 0.001 |
