# Supplementary figures and images for: The prognostic significance of stress hyperglycemic ratio in critically Ill patients with hypertension: A study using the MIMIC-IV database
Source: PLoS One. 2026 Jul 31;21(7):e0352162. doi: 10.1371/journal.pone.0352162 (PMC13426943; doi:10.1371/journal.pone.0352162)

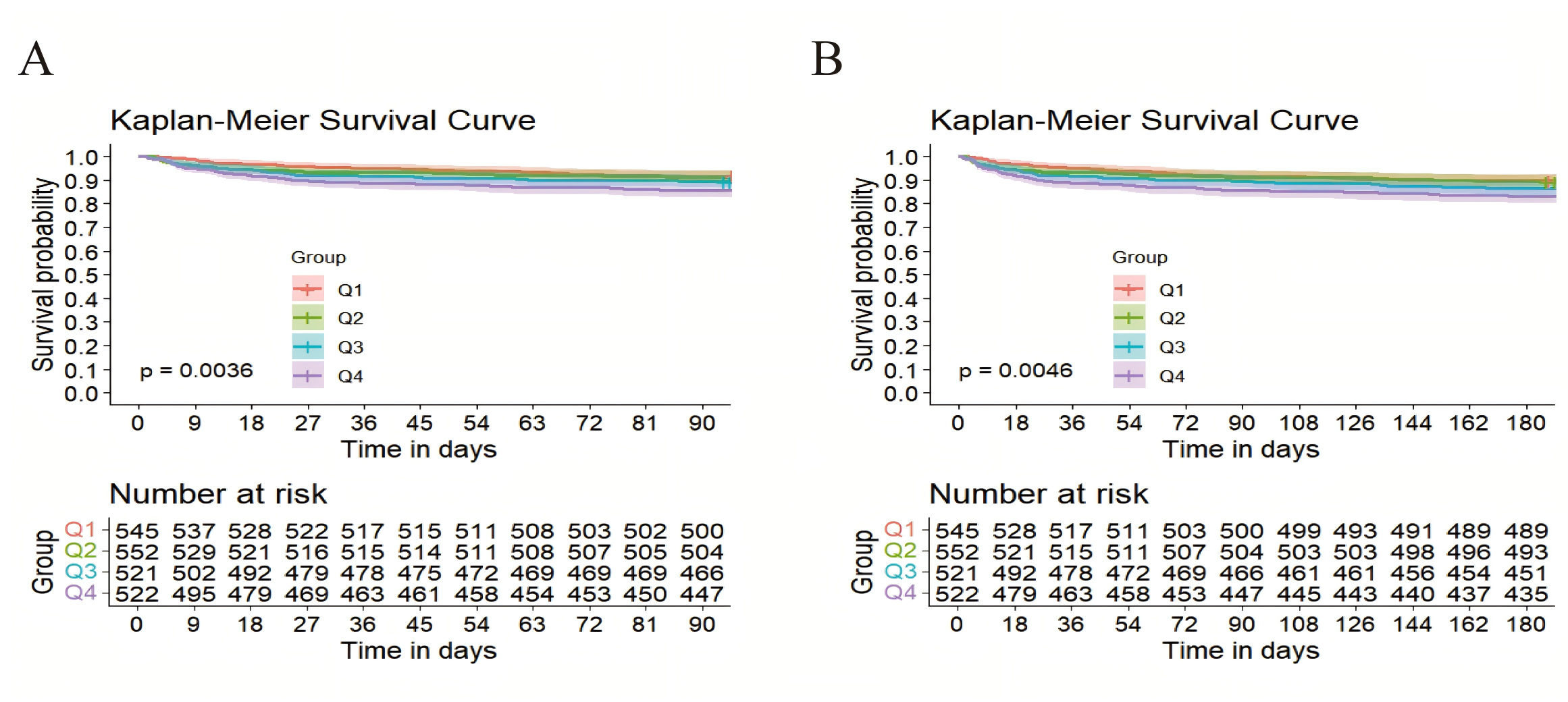

Supplement: S1 Fig — (TIF) [file pone.0352162.s011.tif]

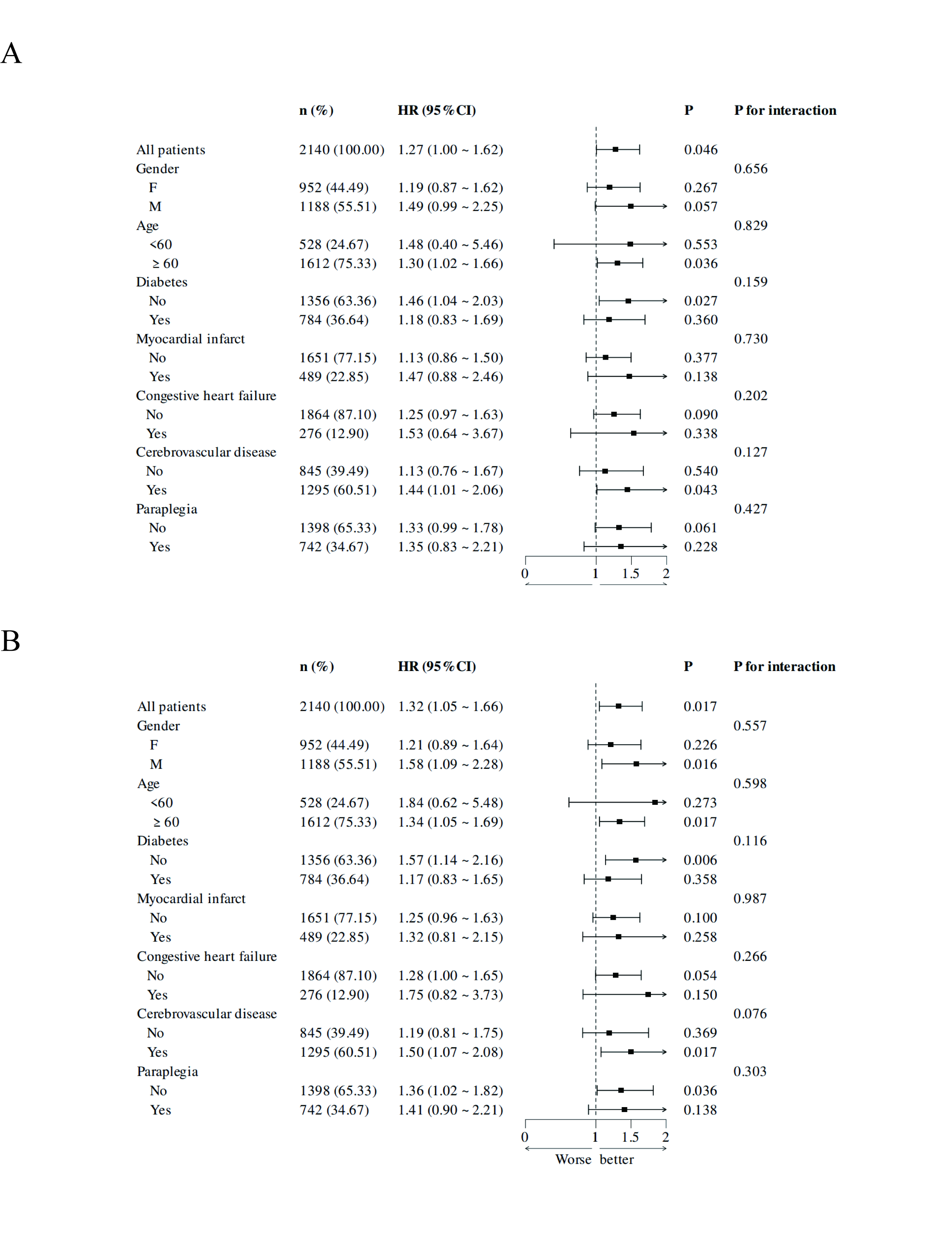

Supplement: S2 Fig — (TIF) [file pone.0352162.s012.tif]
